# Secretor status is a modifier of vaginal microbiota-associated preterm birth risk

**DOI:** 10.1099/mgen.0.001323

**Published:** 2024-12-04

**Authors:** Samit Kundu, Gonçalo dos Santos Correia, Yun S. Lee, Sherrianne Ng, Lynne Sykes, Denise Chan, Holly Lewis, Richard G. Brown, Lindsay Kindinger, Anne Dell, Ten Feizi, Stuart M. Haslam, Yan Liu, Julian R. Marchesi, David A. MacIntyre, Phillip R. Bennett

**Affiliations:** 1 March of Dimes European Prematurity Research Centre, Imperial College London, London, UK; 2 Institute for Reproductive and Developmental Biology, Imperial College London, Hammersmith Hospital Campus, London W12 0NN, UK; 3 Imperial College Healthcare NHS Trust, Parasol Foundation for Women’s Health, London, UK; 4 Institute for Women’s Health, University College London, London, UK; 5 Department of Life Sciences, Imperial College London, London, UK; 6 Glycosciences Laboratory, Department of Metabolism Digestion and Reproduction, Imperial College London, London, UK; 7 Division of Digestive Medicine, Department of Metabolism, Digestion and Reproduction, Faculty of Medicine, Imperial College London, London, UK; 8 Robinson Research Institute, University of Adelaide, Adelaide, Australia

**Keywords:** blood-type antigens, *Lactobacillus*, preterm birth, secretor status, vaginal microbiome

## Abstract

Mutations in the *FUT2* gene that result in a lack of expression of histo-blood group antigens on secreted glycoproteins may shape the vaginal microbiota with consequences for birth outcome. To test this, we analysed the relationship between secretor status, vaginal microbiota and gestational length in an ethnically diverse cohort of 302 pregnant women, including 82 who delivered preterm. *Lactobacillus gasseri* and *L. jensenii* were found to have distinct co-occurrence patterns with other microbial taxa in non-secretors. Moreover, non-secretors with *Lactobacillus* spp. depleted high diversity vaginal microbiota in early pregnancy had significantly shorter gestational length than *Lactobacillus* spp. dominated non-secretors (mean of 241.54 days (sd=47.14) versus 266.21 (23.61); *P*-value=0.0251). Similar gestational length differences were observed between non-secretors with high vaginal diversity and secretors with *Lactobacillus* spp. dominance (mean of 262.52 days (SD=27.73); *p*-*value*=0.0439) or depletion (mean of 266.05 days (SD=20.81); *p*-*value*=0.0312). Our data highlight secretor status and blood-group antigen expression as being important mediators of vaginal microbiota–host interactions in the context of preterm birth risk.

## Data Summary

All sequencing data used in this study have been made available in European Nucleotide Archive (ENA) database (accession numbers: ERR4864561-ERR4865307). Data analysis scripts, processed sequencing data and clinical and demographic variables are publicly available in the following repository: https://github.com/gscorreia89/FUT2, https://zenodo.org/doi/10.5281/zenodo.10777100.

**Impact Statement**

The vaginal microbiome is a risk factor for poor pregnancy outcomes, including preterm birth. Women with high vaginal levels of bacteria called *Lactobacillus crispatus* have reduced risk of preterm birth. In contrast, women with high vaginal bacterial diversity and low levels of *Lactobacillus* have increased vaginal inflammation and are at increased risk of preterm birth. Both vaginal and bacterial cells, as well as proteins that make up vaginal secretions, are decorated by types of sugars called glycans. These can be used as anchor points for bacteria to adhere to and as food sources. In this study, we show that women who lack specific glycans in their vaginal secretions (called blood-type antigens) are more likely to experience a shorter pregnancy gestation if they have a high vaginal bacterial diversity. These findings provide the first evidence that the relationship between the vaginal microbiome and preterm birth risk is likely regulated by the types of glycans produced in vaginal secretions. This provides new understanding of bacterial colonization in the female reproductive tract and highlights glycans as possible targets for therapeutic strategies designed to optimize the vaginal microbiome composition and protect against preterm birth.

## Introduction

Histo-blood group antigens, ABH and Lewis, are carbohydrate sequences found on the surface of a range of cell types and in secretions such as cervicovaginal fluid. These antigens can serve as attachment sites and energy sources for microbial organisms [1,3]. The *FUT2* gene encodes the α(1, 2)-fucosyltransferase enzyme, which adds a fucose residue to the terminal galactose on a type 1 glycan precursor forming the H antigen. Four mutations in the *FUT2* gene have been identified (alleles *se^302^*, *se^385^*, *se^428^* and *se^571^*) that result in non-secretion of these antigens from mucosal surfaces, in homozygotes. Up to 20% of human populations express this non-secretor phenotype [4,7]. Non-secretors will present as either Le(a+b-) or Le(a-b-), depending upon the presence of mutations in their *FUT3* gene. Secretor status has been associated with both increased and decreased susceptibility to a range of bacterial and viral pathogens as well as gut microbiota composition [8,15], such as Norovirus, *Streptococcus pneumoniae* or *Haemophilus influenzae*.

The vaginal microbiome plays an important role in influencing pregnancy outcome. Dominance of the vaginal niche by *Lactobacillus* species has been widely reported to associate with healthy, term pregnancy [16,17]. In contrast, high-diversity microbial communities depleted of *Lactobacillus* species and enriched with bacteria associated with BV, a clinical syndrome of vaginal discharge and odour characterized by polymicrobial overgrowth-associated bacteria, increase the risk of adverse pregnancy outcomes including miscarriage and preterm birth [18,20]. Preterm birth represents a significant global health burden. It is the leading cause of death in children under age 5 and associated with serious short- and long-term morbidities in survivors [21,23]. Significant risk factors for spontaneous preterm birth in our populations include a history of late miscarriage or preterm birth and previous cervical excisional treatment, an operation to treat cervical pre-cancer [24,25]. Both a history of miscarriage and cervical pre-cancer have been linked with reduced vaginal dominance by *L. crispatus* and enrichment for *L. iners* or BV-associated bacteria [16,17, 26, 27].

In a recent study of 300 pregnant women (of whom 28 experienced preterm birth), maternal secretor status was reported as an independent risk factor for preterm delivery [28]. Specific human milk oligosaccharides (HMOs) in blood and urine have also been recently associated with vaginal microbiota in a small cohort (*n*=60) of women presenting with threatened preterm labour [29]. We postulated that the composition of the vaginal microbiota in pregnancy might be influenced by maternal secretor status and that this may influence pregnancy duration. To characterize the effect of secretor status on the vaginal microbiome and gestational length, we sequenced the second exon of the *FUT2* gene to infer secretor status and undertook metataxonomic analysis of vaginal samples collected longitudinally from a cohort of 302 pregnant women, of which 82 delivered preterm.

## Methods

### Patient sampling

Recruitment and sampling were performed at Imperial College Healthcare NHS Trust Hospitals (Queen Charlotte’s and Chelsea and St Mary’s Hospitals), London, UK, at Chelsea and Westminster Hospital (NHS Trust, London, UK), University College London Hospital (NHS Foundation Trust, London, UK) and the Royal Infirmary of Edinburgh, Scotland, UK. Eligibility criteria included singleton pregnancies, with and without risk factors for preterm birth. Exclusion criteria included women under 18 years of age, those who had sexual intercourse within 72 h of sampling, vaginal bleeding in the preceding week, antibiotic use in the preceding 2 weeks, multiple pregnancies, HIV or hepatitis C positive status. Detailed maternal clinical metadata and birth outcome data were collected for all participants. Cervicovaginal swab samples were collected from the posterior fornix using BBL CultureSwab MaxV Liquid Amies swabs (Becton, Dickinson and Company, Oxford, UK) at up to three timepoints throughout pregnancy: early (63–131 days), mid (133–178 days) and late (183–251 days). Swabs were immediately placed in Amies transport media and stored at −80 °C.

### Determination of secretor status

Extraction of DNA from vaginal swabs was performed as previously described [30]. Individuals were genotyped by sequencing the coding part of exon 2 of the *FUT2* gene. The exon was amplified using primers 5′-CCATATCCCAGCTAACGTGTCC-3′ and 5′-GGGAGGCAGAGAAGGAGAAAAGG-3′ [31] and the amplicons sequenced with the PacBio Sequel system. Primer sequences were removed from the CCS reads using Dada2 [32] and the trimmed sequences were mapped to a human reference FUT2 sequence (derived from HG38) using Minimap [33]. To limit the effect of any reference bias, we generated consensus sequences using the ‘ALT’ allele from the mapping, and reads were remapped to this sequence. Finally, variants were called using Freebayes [34] and phased using WhatsHap [35].

Individuals that were homozygous for any one of the four known non-secretor mutations (se302 (P101L, rs200157007), se385 (I129F, rs1047781), se428 (W413X, rs601338) and se571 (R191X, rs18000028)) were inferred to be phenotypically non-secretor [4,6,36]. Heterozygotes and wild-type homozygotes were classified as secretors [37].

### Sequencing of 16S rRNA gene amplicons and assembly

The V1-V2 hypervariable regions of bacterial 16S rRNA genes were amplified with using the forward primer set (28f-YM) consisting of a mixture of the following primers mixed at a 4 : 1 : 1 : 1 ratio: 28F-Borrellia GAGTTTGATCCTGGCTTAG; 28F-Chlorflex GAATTTGATCTTGGTTCAG; 28F-Bifido GGGTTCGATTCTGGCTCAG; 28F GAGTTTGATCNTGGCTCAG. The reverse primer consisted of 388R TGCTGCCTCCCGTAGGAGT [38]. Sequencing was performed on the Illumina MiSeq platform. Primer sequences were trimmed using Cutadapt [39]; quality control was performed using FastQC [40] and amplicon sequence variants counts per sample were calculated with the Qiime2 pipeline [41]. We used DADA2 [32] for denoising and taxonomically classified sequences to species or genera level using a bespoke Naïve Bayes classifier [42] trained on the silva SSU (version 138) reference database [43], using the Qiime2 feature–classifier interface.

### Data analyses

Generalized linear mixed effects gamma regression models for each timepoint in pregnancy were used to regress gestational length (in days) on secretor status and the vaginal microbiome (*Lactobacillus* status or community state types (CST)) as well as age, BMI, previous preterm birth (PTB)/mid-trimester loss (MTL), cervical stitch and previous cervical excisional treatment in glmmTMB [44]. We applied a reflection transformation to the gestational length prior to fitting the gamma generalized linear mixed effects modelling (GLMM) as this variable was negatively skewed. Model diagnostics were examined with scaled residuals simulated from the fitted models using DHARMa [45]. We used the emmeans package to calculate the estimated marginal means from the models to look for statistically significant comparisons between groups [46]: this *post-hoc* method automatically adjusts for multiple comparisons.

We retained taxa that occurred at greater than 0.5% abundance in two or more samples. Differential abundance analyses were performed using ALDEx2 to compare taxon abundances in the vaginal microbiome in early pregnancy between secretors and non-secretors [47,48]. This method uses a compositionally robust approach to perform differential abundance analyses (DAA): microbiome count data are considered compositional and thus have to be transformed to permit analysis by multivariate statistics [49]. Count data were transformed with the centred log-ratio (CLR), and we ran 1000 Monte Carlo instances to estimate effect sizes and perform a Welch’s *t*-test to compare our two conditions. We also analysed the compositional differences between phenotypes using principal component analysis (PCA): zeros in the count data were imputed using a Bayesian multiplicative approach in the zCompositions package [50] followed by CLR transformation. To test whether the centroids of the two secretor status groups differ, we performed a Permutational multivariate analysis of variance (PERMANOVA) on the CLR-transformed distance matrix (after checking for homogeneity of dispersion) using the Vegan package [51].

Finally, we used BAnOCC to infer co-occurrence networks for the microbial taxa observed in secretors and non-secretors in early pregnancy [52]. This programme uses a Bayesian framework to analyse compositional covariance. We ran the MCMC for 200 000 iterations and 8 chains to reach convergence, with the ‘adapt_delta’ parameter set to 0.9 to reduce the number of divergent transitions. We used a 95% credible interval to identify significant correlations. Qgraph and ggraph were used to draw the networks based on these BanOCC-derived adjacency matrices and to calculate the Expected Influence (EI) network statistic, which is a measure of degree centrality for signed networks [53,54].

## Results and discussion

### Distribution of non-secretors in our cohort

A total of 302 women were included in our study (Table S1, available in the online Supplementary Material), of whom 82 experienced spontaneous preterm birth before 37 weeks (259 days) of gestation (Table S2). The ethnic distribution of the cohort was similar to the expected background prevalence within the clinical population. In total, 83 women were identified as non-secretors and 202 as heterozygous at these loci. Although nine women were heterozygous at multiple non-secretor loci, we did not identify any who were multiply homozygous. All four known non-secretor mutations were identified in our cohort. The *se^428^* nonsense mutation accounted for 94.32% of the heterozygotes and 93.98% of the non-secretor homozygotes and was found in individuals from all ethnicities except in the mixed/other ethnicity category. The remaining mutations had more restricted distributions: four women of Asian ethnicity were homozygous for the *se^385^* non-secretor allele, with three heterozygous individuals of Asian and two of white descent. Only one *se^302^* homozygote was identified in a woman of Asian ethnicity (five other women, of similar ethnicity, were heterozygous). We did not identify any homozygotes of *se^571^*. These patterns are consistent with previously documented distributions of non-secretor genetic diversity [5,7]. Overall, 27.48% of the cohort were identified as non-secretor with the highest proportion in white women, 29.41%, compared with 25.81 and 26.82% in black and Asian ethnicities, respectively. For all subsequent analyses, secretor homozygotes were combined with heterozygotes into a single group since they are phenotypically similar [37].

### Lactobacilli are more refractory to co-colonization in non-secretors

Vaginal microbiota were classified into one of five CST using the VALENCIA classifier [55]. The most prevalent CST observed in our cohort was CST 1 (majority *L. crispatus*) in 45.8, 49.24 and 45.80% of the patients at early (9–19 weeks), mid (19–26 weeks) and late (26–36 weeks) gestation, respectively, followed by CST 3 (majority *L. iners*) in 29.01, 24.62 and 26.89%; CST 4 (diverse) in 14.12, 12.88 and 13.87%; CST 5 (majority *L. jensenii*) in 7.25, 8.33 and 10.08%; and CST 2 (majority *L. gasseri*) in 3.82, 4.92 and 3.36%.

**Fig. 1.**
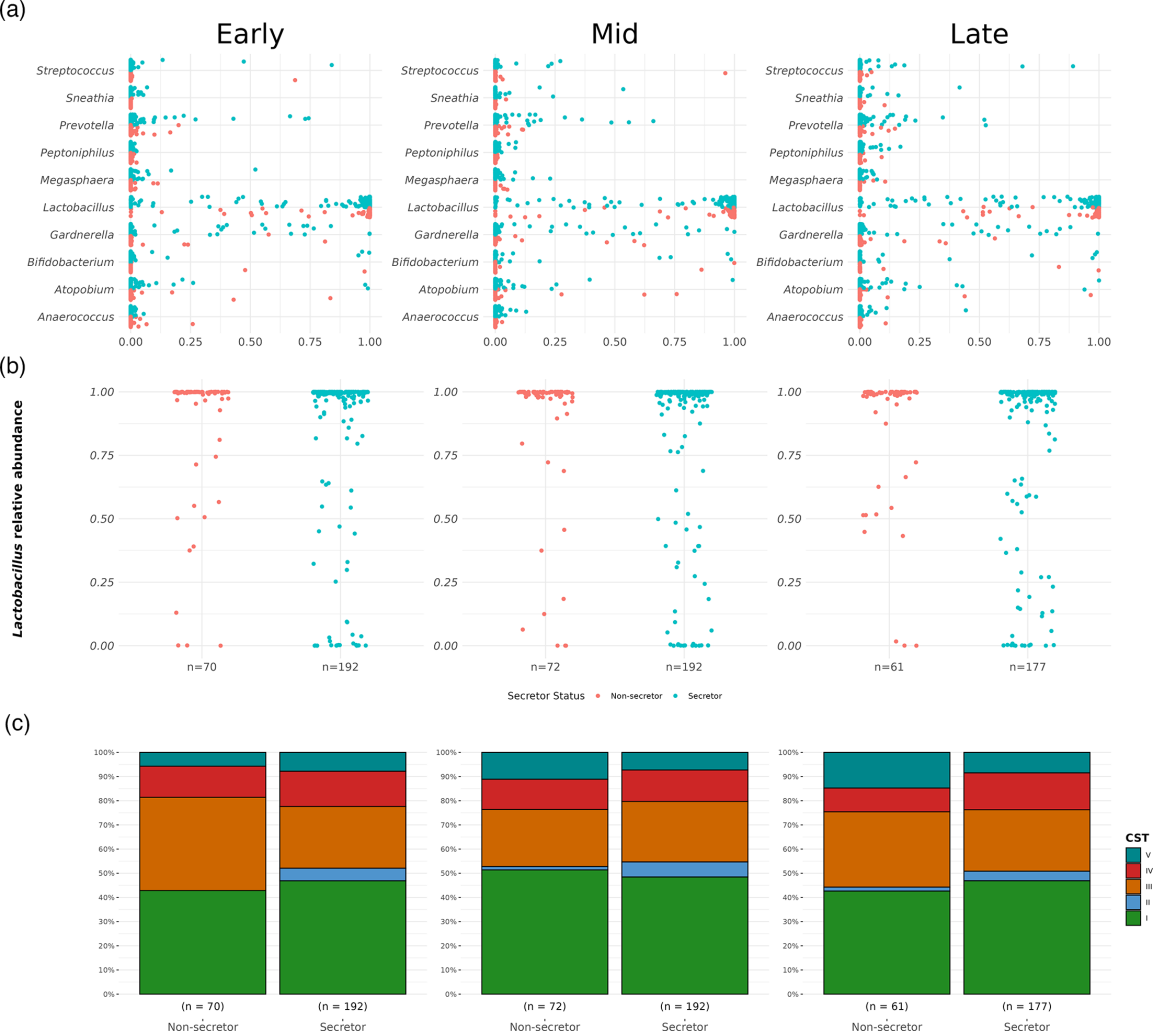
Vaginal microbiota composition in secretors and non-secretors.(**a**) Distribution of the top 10 most abundant bacterial genera (in descending alphabetical order) in the vaginal microbiota of secretors and non-secretors in early, mid and late pregnancy. (**b**) The distribution of the proportion of *Lactobacillus* in secretors and non-secretors in early, mid and late pregnancy was similar. (**c**). Frequencies of each CST through pregnancy in secretors and non-secretors.

In samples taken during early pregnancy, prior to any interventions, we observed a similar prevalence of major bacterial genera across secretor and non-secretor women, including those classically associated with BV, e.g. *Gardnerella*, *Atopobium, Anaerococcus* and *Sneathia* (Fig. 1a). As expected, most women had a high relative abundance of *Lactobacillus*, though more variation was observed in secretors (Fig. 1b). At species level, an enrichment for CST 3 in non-secretors and CST 4 in secretors was observed, but these differences were not statistically significant (Fisher’s Exact test *P*-value=0.12, Fig. 1c).

We observed no difference in bacterial diversity (Shannon index) between non-secretors and secretors in early pregnancy (Wilcoxon rank-sum test, *P*-value=0.949) nor any differential classification in PCA (PERMANOVA *P*-value=0.537). DAA did not identify any significant differences in relative species abundance based on secretor phenotype (Fig. S1). However, the low prevalence of *Lactobacillus*-depleted patients (20.61% of all early pregnancy samples) in our cohort may have limited our power to detect such associations. Low prevalence of *Lactobacillus*-depleted vaginal communities is likely attributable to increased relative abundance of *Lactobacillus* species in the vaginal microbiome during pregnancy and is consistent with our clinical population: mostly women with European ancestry who, compared with other ethnicities, have higher prevalence of *Lactobacillus*-dominated vaginal microbiomes [30,56]. A recent study of 60 women presenting with suspected preterm labour reported positive associations between specific urinary and blood HMOs, and some vaginal taxa and clinical outcomes including preterm birth [29]. We have not found robust evidence for an impact of secretor status on vaginal microbial composition (i.e. CST prevalences) or relative abundance of specific taxa in our study. However, it is difficult to compare our findings to those of Pausan *et al*. [29] given that over half of their patient cohort received tocolysis to inhibit uterine contractions and prevent preterm birth (only a small number of women in their study cohort experienced preterm birth, *n*=11), and we have not measured serum or urinary HMOs.

**Fig. 2.**
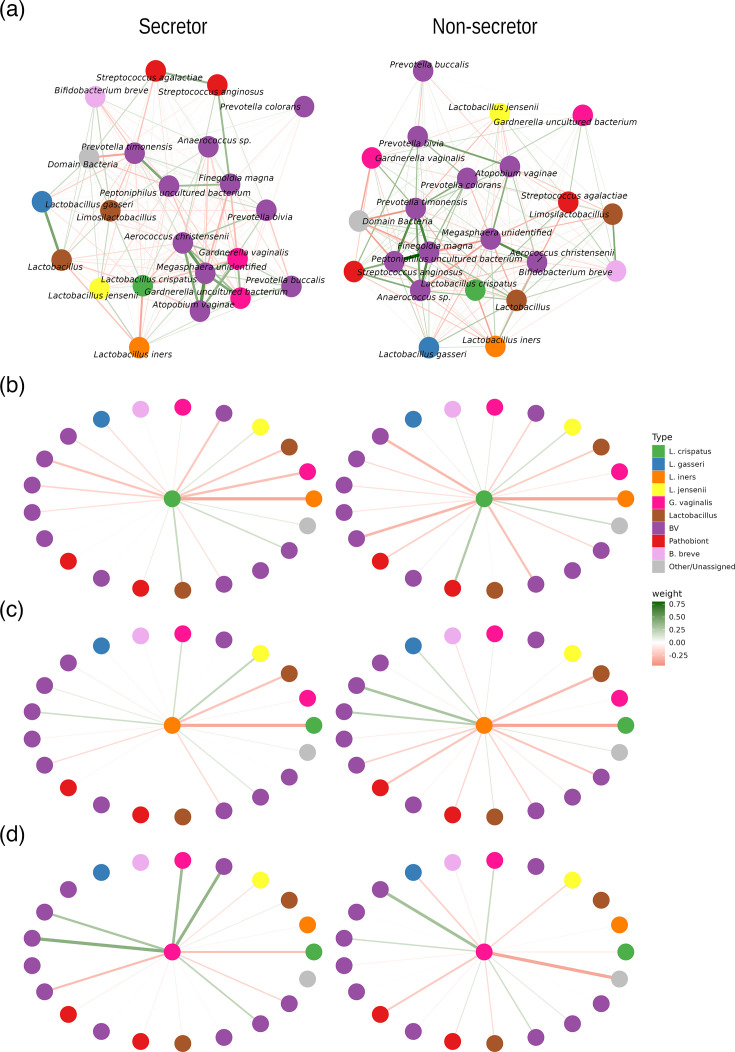
Co-occurrence of vaginal microbiota in early pregnancy in the context of secretor status. (**a**) Co-occurrence networks of early pregnancy vaginal microbiota in secretors and non-secretors. Nodes represent bacterial taxa detected at >0.5% relative abundance in two or more samples and have been grouped into *L. crispatus*, *L. gasseri*, *L. iners*, *L. jensenii*, other *Lactobacillus*, BV-associated bacteria, pathobionts and others, as defined previously. Statistically significant edges, indicating correlations, are coloured and weighted according to their *r* value: red (negative correlation); green (positive correlation). In both secretors and non-secretors, many positive associations were observed between BV-associated microbes. (**b**) Star diagrams (derived from the network analyses) centred on the *L. crispatus*, *L. iners* and *G. vaginalis* nodes indicate *Lactobacillus* species, especially *L. crispatus*, tend to be more negatively correlated with other bacteria in the vaginal microbiota of both secretors and non-secretors, compared with *G. vaginalis* and other BV-associated bacteria.

To investigate whether vaginal microbial community structure might differ between secretors and non-secretors, we compared co-occurrence networks inferred from correlation matrices based on the 16S relative abundance data (Fig. 2) as previously described [57]. For this analysis, non-*Lactobacillus* taxa were classified as ‘pathobionts’, ‘BV-associated’ or ‘other’ using definitions from Wijgert *et al*. [58]. In the non-secretor microbial network, nodes representing BV-associated bacteria tended to be connected via positive edges, reflecting positive correlations among these microbes (Fig. 2a). The analysis of node importance, using EI as a metric [59], confirmed this pattern, i.e. that in the non-secretor network, nodes with the most positive EI scores were BV-associated bacteria and pathobionts, such as *Prevotella timonensis*, *Prevotella colorans*, *Prevotella bivia*, *Anaerococcus* sp., *Peptoniphilus* and *Finegoldia magna* (Fig. S2). The secretor network displayed slight changes in these patterns, with negative EI scores for *Prevotella timonensis*, *Prevotella colorans* and *Peptoniphilus*, lower positive EI scores for *Prevotella bivia* and positive score for *Gardnerella vaginalis*. These patterns mirror previous studies showing that these microbes establish interactions based on mutualistic relationships and suggest that secretor status could impact bacterial interactions. *L. crispatus*, *L. jensenii* and *L. iners* were among the most negatively scored nodes for EI in both networks. A greater proportion of negative edges from these lactobacilli to other BV-associated microbes was observed, especially with *L. crispatus* (Figs 2b and S2). These results suggest that *Lactobacillus* species, particularly *L. crispatus*, are more refractory to co-colonization in non-secretors where they may offer a greater ‘protective’ effect via competitive exclusion. These findings are consistent with comparative genomics studies that have identified differences in carbohydrate degradation enzyme repertoire between *L. crispatus* and an increased prevalence of a 3-fragmented glycosyltransferase gene in *L. crispatus* strains isolated from dysbiotic vaginal microbiota [60]. This highlights bacterial cell surface glycoconjugates and carbohydrate-binding proteins as potentially important mediators of vaginal microbiota–host interactions [60].

### Non-secretors with *Lactobacillus*-depleted vaginal microbiota experience shorter gestational length

**Fig. 3.**
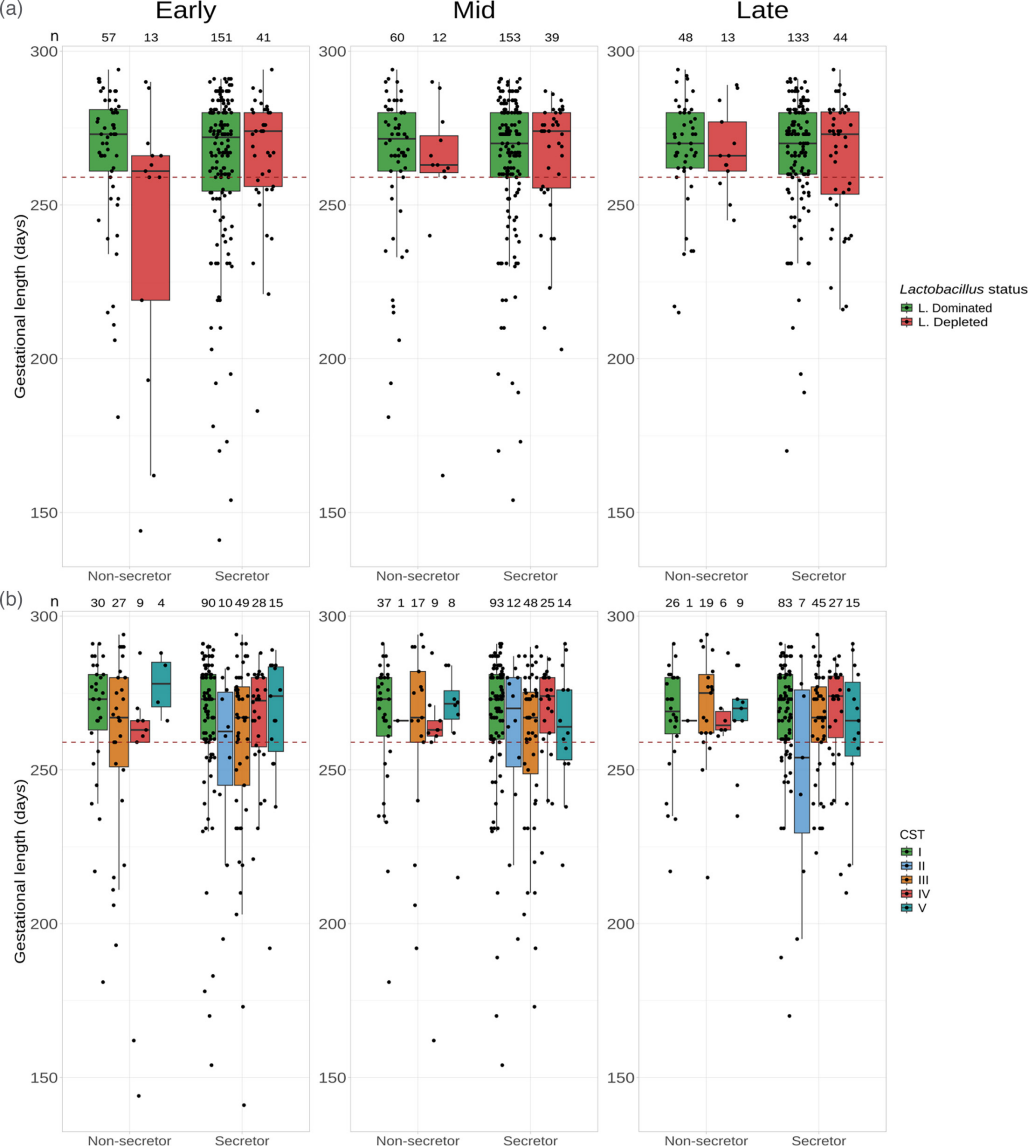
The association between secretor status, vaginal microbiota and gestational length (days). A red dashed line highlights the 37 weeks (259 days) threshold for prematurity. (**a**) The distribution of gestational length by secretor status and vaginal microbiome shows that non-secretors with *Lactobacillus*-depleted microbiomes in early pregnancy tend to have shorter gestation, but this relationship disappears by mid-pregnancy. Sample sizes are presented at the top of plot. (**b**) Classifying the microbiome by CST rather than by *Lactobacillus* status indicates that reduced gestation in *Lactobacillus*-depleted non-secretors is mainly driven by women with CST 3 and high diversity CST 4 microbiomes. Sample sizes are presented in Table S3.

Based on the distribution of *Lactobacillus* abundance (Fig. 1c) and previous studies [61,62], we classified the vaginal microbiome into *Lactobacillus*-dominated (>90% *Lactobacillus* spp.) and *Lactobacillus*-depleted states (<90%). Non-secretors with a *Lactobacillus*-depleted microbiome in early pregnancy had a shorter mean gestational length, 241.54 days (sd=47.14) compared with *Lactobacillus*-dominated non-secretors and 266.21 days (sd=23.61, *P*-value*=*0.0251, Welch’s *t*-test with Tukey Honestly Significant Difference (HSD) *post-hoc* correction), and to both *Lactobacillus*-depleted and *Lactobacillus*-dominated secretors, 266.05 (sd=20.81, *P*-value=0.0312) and 262.52 (sd=27.73, *P*-value=0.0439) days, respectively (Fig. 3a). However, the association between *Lactobacillus* status in non-secretors on gestational outcome was diminished by mid-pregnancy. To confirm these observations, for each sampling timepoint, we modelled gestational length on secretor status and the vaginal microbiome (*Lactobacillus* status) together with BMI, age, cervical cerclage status and previous pregnancy history, i.e. cervical excisional treatment (LLETZ) and a combined previous PTB and/or MTL covariate using GLMM with ethnicity as a random effect (Table 1). As expected, previous pregnancy history was a significant explanatory variable of gestational length (Table S4). Comparison of the GLMMs with generalized linear models (GLM) showed similar model fits (e.g. Akaike information criterion (AIC) 2199.407 and 2201.407 for the GLM and GLMM, respectively, for the early pregnancy modelling) indicating that ethnicity was not a significant factor in the model. However, the early pregnancy GLMM showed that the secretor status and *Lactobacillus* status interaction term is a significant explanatory variable of gestational length (*P*-value=0.0108). This significance disappeared by mid-pregnancy supporting our earlier observation that non-secretors with a *Lactobacillus*-depleted vaginal microbiome in early pregnancy, but not mid-pregnancy, are associated with shorter gestational lengths. *Post-hoc* testing using the estimated marginal means confirmed this significant comparison (*P*-value=0.0186, secretor versus non-secretors with *Lactobacillus*-depleted microbiome). No statistically significant difference between secretors and non-secretors was found for pregnancies with a *Lactobacillus*-dominated microbiome (*P*-value=0.3145). No significant difference between *Lactobacillus*-dominated and -depleted secretors was found (*P*-value=0.0973). To ensure the robustness of these findings to the choice of *Lactobacillus* relative abundance threshold (90%) to assign samples to *Lactobacillus*-dominated or *Lactobacillus*-depleted categories, a sensitivity analysis was performed by varying the threshold from 25 to 90%. The secretor and *Lactobacillus* status interaction term was consistently statistically significant at *α*=0.05 for all thresholds above 50% (Fig. S3).

**Table 1.** Unstandardized coefficients (β) and 95% confidence intervals (CI) from gamma GLMM of gestational length (in days) over three time points in pregnancy (with ethnicity as a random effect). Early, mid- and late pregnancy GLMMs using *Lactobacillus* status (dominated/depleted), as well as an early CST GLMM, which replaces the *Lactobacillus* covariate with CST. Secretors with *Lactobacillus*-dominated microbiota (or CST 1) are baseline in the model

	Early	Mid	Late	Early (CST)
	β (95% CI)	β (95% CI)	β (95% CI)	β (95% CI)
Intercept	3.375 (2.609, 4.142)‡	3.053 (2.302, 3.804)‡	2.894 (2.140, 3.649)‡	3.263 (2.462, 4.064)‡
Maternal age	0.003 (−0.013, 0.019)	0.010 (−0.006, 0.026)	0.013 (−0.003, 0.029)	0.006 (−0.011, 0.022)
BMI	−0.013 (−0.031, 0.006)	−0.007 (−0.024, 0.010)	−0.006 (−0.023, 0.012)	−0.012 (−0.030, 0.007)
Previous PTB§/MTL¶	0.541 (0.353, 0.729)‡	0.339 (0.154, 0.524)‡	0.324 (0.141, 0.506)‡	0.527 (0.330, 0.724)‡
Cervical stitch	0.361 (0.171, 0.552)‡	0.414 (0.248, 0.580)‡	0.224 (0.056, 0.392)†	0.366 (0.175, 0.557)‡
Previous cervical excisional treatment	−0.138 (−0.330, 0.054)	−0.199 (−0.387, –0.011)*	−0.129 (−0.317, 0.059)	−0.152 (−0.348, 0.044)
Secretor ×*Lactobacillus*	0.617 (0.143, 1.092)*	0.268 (−0.201, 0.737)	0.078 (−0.378, 0.534)	
Secretor ×CST 2				na(low sample size)
Secretor×CST 3				0.264 (−0.172, 0.699)
Secretor×CST 4				0.467 (−0.125, 1.060)
Secretor×CST 5				−0.348 (−1.149, 0.454)

*P<0.05.

†*P*<0.01.

‡*P*<0.001.

§Preterm birth.

¶Mid-trimester loss.

When vaginal microbiomes were classified into CSTs, non-secretors with CST 4 in early pregnancy had shorter gestations, mean (±sd) of 242.11 (51.41) days, respectively, compared with CST 1 (266.87 days (23.95), Welch’s *t*-test *P*-value*=*0.0195), but the statistical significance did not withstand correction for multiple testing (Fig. 3b, Welch’s *t*-test with Tukey HSD *post-hoc* correction *P*-value*=*0.3165). A similar pattern was observed with CST 3 (mean of 28.34, 259.96) and CST 5 (mean of 277.50 days); however, this did not reach statistical significance, and the sample size of CST 5 was limited to *n*=4. No non-secretors with CST 2 were observed in early gestation. By contrast, gestational length in secretors was broadly similar across all CSTs: mean gestational length (±sd) of 265.81 (26.07), 254.70 (28.59), 258.12 (30.96), 266.25 (16.73) and 265.07 (24.97), respectively, in CST 1, 2, 3, 4 and 5 (no statistically significant differences, with or without Tukey’s *post-hoc* correction). When modelling gestational length with early pregnancy CST (Table S7), the analysis of deviance does not support an interaction between CST and secretor status (*P*-value=0.203), neither in mid- or late pregnancy. The estimated marginal means comparisons did not identify statistically significant differences in gestational length between secretors and non-secretors with CST 4 or between non-secretors with CST 1 and 4 (*P*-value=0.187 and *P*-value=0.2568, respectively). The previous GLMM models used to assess the interaction between *Lactobacillus*-dominated or *Lactobacillus*-depleted and secretor status were repeated using two alternative *Lactobacillus* status assignment criteria based on the VALENCIA CST classification. In the first criteria, all samples classified as CST 4 were directly assigned to *Lactobacillus* depleted. In the second, the *Lactobacillus*-depleted category was further curated by assigning as *Lactobacillus* dominated all sub-CSTs IV-C3, where *Bifidobacterium breve* is the predominant species, and edge cases of sub-CST IV-C0, where *Lactobacillus* species other than *L. crispatus*, *L. iners*, * L. gasseri* or *L. jensenii* were observed to have the highest relative abundance. This latter criterion was implemented to avoid confounding the associations through the pooling of *B. breve* and other *Lactobacillus* species with BV-like CST 4 bacterial communities, as VALENCIA does by default. The term for the interaction between secretor status and *Lactobacillus* status was not statistically significant in the models fitted using both criteria (Tables S5 and S6). However, in a GLMM model using the relative abundance of *Lactobacillus* genera counts as a continuous covariate, a significant interaction between secretor status and *Lactobacillus* abundance was observed (*P*-value*=*0.024, Table S5). Altogether, these observations point towards a nuanced interaction between secretor status and bacterial communities dominated by *Lactobacillus* species. A major limitation of this present study is the small sample size, especially the number of non-secretor *Lactobacillus*-depleted women, where most of the statistically significant associations were detected. The sample sizes for CST 4 in non-secretors also precluded robust direct comparisons between VALENCIA individual sub-CSTs.

Preterm premature rupture of the membranes (PPROM) precedes approximately 30% of all preterm birth cases and has been associated with ascending infection in the vagina [19,63]. Our findings are consistent with a previous study by Lurie *et al*. [64] who identified a significantly elevated proportion of non-secretors in a cohort of 28 PPROM patients compared with term controls. Caldwell *et al*. [28] also reported maternal secretor status as a potential biomarker for prematurity. Our data supports these findings but demonstrates that this relationship is likely shaped by the vaginal microbiota in early pregnancy. Dominance of the vaginal niche by *Lactobacillus* spp. is considered to be a hallmark of vaginal health due to the role that these species play in preventing colonization of other microbes through the promotion of a hostile, acidic mucosal environment enriched by bacteriocins and other antimicrobial compounds [65,66]. Our results indicate that this protective role extends into pregnancy but is nuanced by host secretor status. Studies identifying an association between vaginal *Lactobacillus* spp. depletion and increased risk of preterm birth [56,67,71] also emphasize that high diversity vaginal bacterial communities do not always result in preterm birth (as is the case of the secretors). An enhanced inflammatory response, previously observed in non-secretors [72], may provide a mechanism linking gestational length to secretor status and vaginal microbiota, but this requires further investigation. It is possible that ABO blood groups influence the relationship between secretor status and the microbiota by affecting terminal glycan structures. To address this, we incorporated ABO status into our models and observed that there may be evidence of shorter gestational length in blood group B women compared with blood group A, though this was only observed at mid- and late-pregnancy (Table S8).

Early developmental stages in pregnancy have lasting effects on pregnancy gestation and outcome. Stout and colleagues showed that, in a predominantly African-American cohort, early gestation is an ecologically important time for events that predict subsequent term and preterm birth [73]. Similarly, Tabatabaei *et al*. [74] identified an association between *Lactobacillus*-depleted vaginal microbiota in early pregnancy and increased risk of early preterm birth in a cohort of Canadian women of predominantly white European origin. Our data indicate that the relationship between *Lactobacillus* depletion and reduced gestational length in non-secretors changes as pregnancy progresses which also points towards the microbiota–host interactions in early pregnancy being particularly key in shaping preterm birth risk. *Lactobacillus* depletion is also a risk factor for miscarriage, which collectively suggests a potential effect of the vaginal microbiota upon decidual function and placentation [18,75].

## Conclusion

This study reports that women who are non-secretors with *Lactobacillus*-depleted vaginal microbiomes are at increased risk of shorter gestational length. Stratification of women based on secretor status and vaginal microbiota composition could allow a more targeted intervention of ‘at-risk’ pregnancies, although these results should be verified with larger numbers of *Lactobacillus*-depleted samples. We find evidence of a more ‘protective’ role of *Lactobacillus* species, especially *L. crispatus*, in non-secretors. This supports the evolving concept that *L. crispatus* offers optimal protection against preterm birth and is a candidate for development of live biotherapeutic therapies during pregnancy.

## supplementary material

10.1099/mgen.0.001323 Uncited Supplementary Material 1.

## Abbreviations

CLR: centred log-ratio
CST: community state types
DAA: differential abundance analyses
GLM: generalized linear models
GLMM: generalized linear mixed effects modelling
HMOs: human milk oligosaccharides
MTL: mid-trimester loss
PCA: principal component analysis
PPROM: preterm premature rupture of the membranes
PTB: preterm birth
