# Supplementary material for: Secretor status is a modifier of vaginal microbiota-associated preterm birth risk
Source: Microb Genom. 2024 Dec 4;10(12):001323. doi: 10.1099/mgen.0.001323 (PMC11616779; doi:10.1099/mgen.0.001323)
Supplement: Uncited Supplementary Material 1. [file mgen-10-01323-s001.pdf]

Supplementary Information

Secretor status is a modifier of vaginal microbiota-associated preterm birth risk

Samit Kundu[1+], Gonçalo dos Santos Correia[1,2+], Yun S Lee[1], Sherrianne Ng[1,2], Lynne Sykes[1,2], Denise Chan[2], Holly Lewis[2], Richard G Brown[2], Lindsay Kindinger[2,3], Anne Dell[1,4], Ten Feizi[1,5], Stuart M Haslam[1,4], Yan Liu[1,5], Julian R Marchesi[1,6], David A MacIntyre[1,2,7*] and Phillip R Bennett[1,2]

[1]March of Dimes European Prematurity Research Centre, Imperial College London

[2]Institute for Reproductive and Developmental Biology, Imperial College London, Hammersmith Hospital Campus, London W12 0NN, United Kingdom

[3]Institute for Women's Health, University College London, London, UK

[4]Department of Life Sciences, Imperial College London, London, UK

[5]Glycosciences Laboratory, Department of Metabolism Digestion and Reproduction, Imperial College London, London, UK

[6]Division of Digestive Medicine, Department of Metabolism, Digestion and Reproduction, Faculty of Medicine, Imperial College London, UK.

[7]Robinson Research Institute, University of Adelaide, Australia

[+]These authors contributed equally to this work

*Correspondence to Prof. D.A. MacIntyre (d.macintyre@imperial.ac.uk)

Supplementary Figures

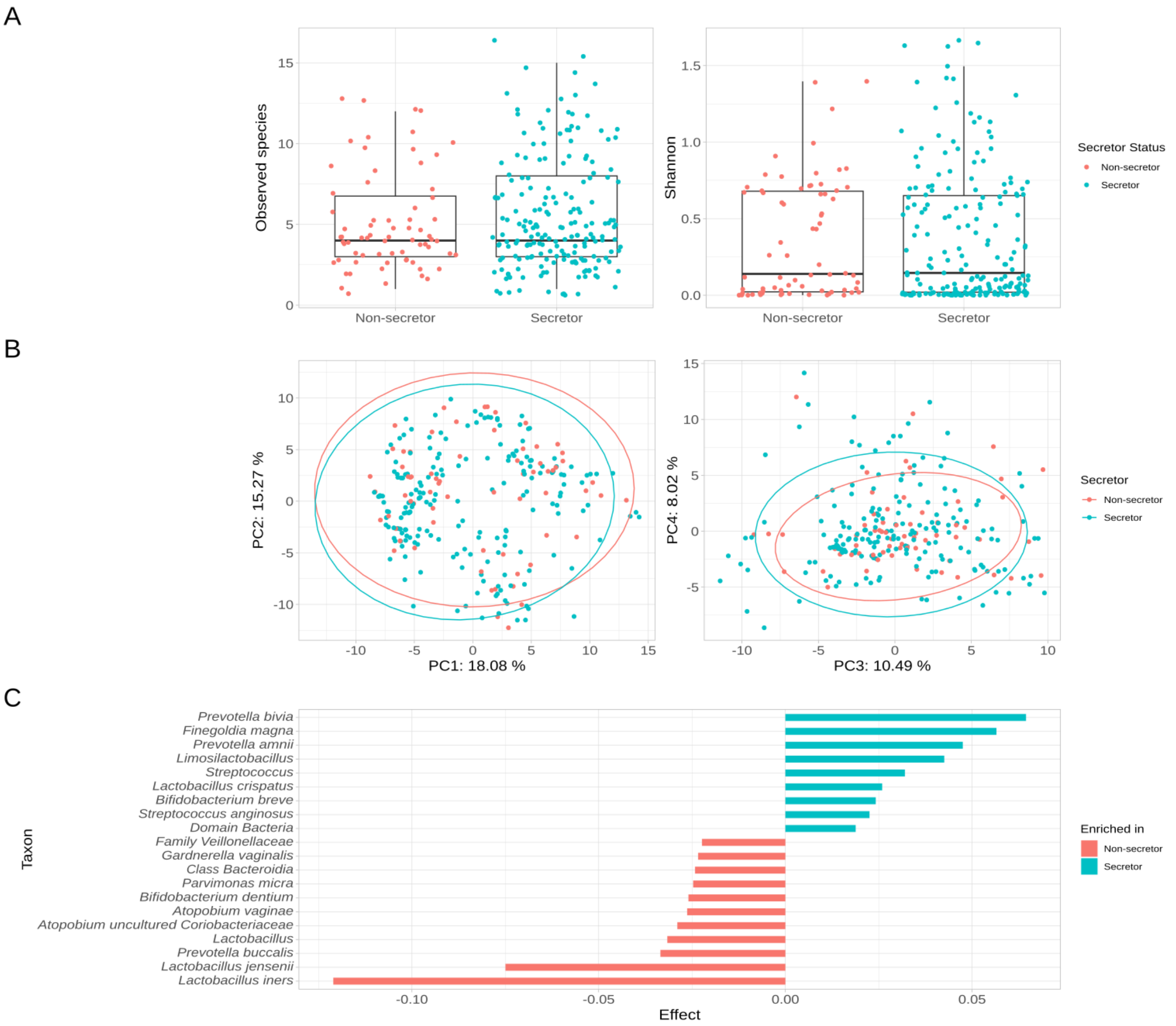

Figure S1. Secretors and non-secretors have similar vaginal microbiota profiles in early pregnancy. (a) Distribution of diversity estimates of the vaginal microbiota in secretors and non-secretors in early pregnancy. **(b)** PCA of the vaginal microbial community in early pregnancy shows no significant difference (first four principal components plotted). **(c)** Differential Abundance Analyses indicated only small and non-significant differences between secretors and non-secretors in the top 20 microbial taxa by effect size, in the early pregnancy microbiota.

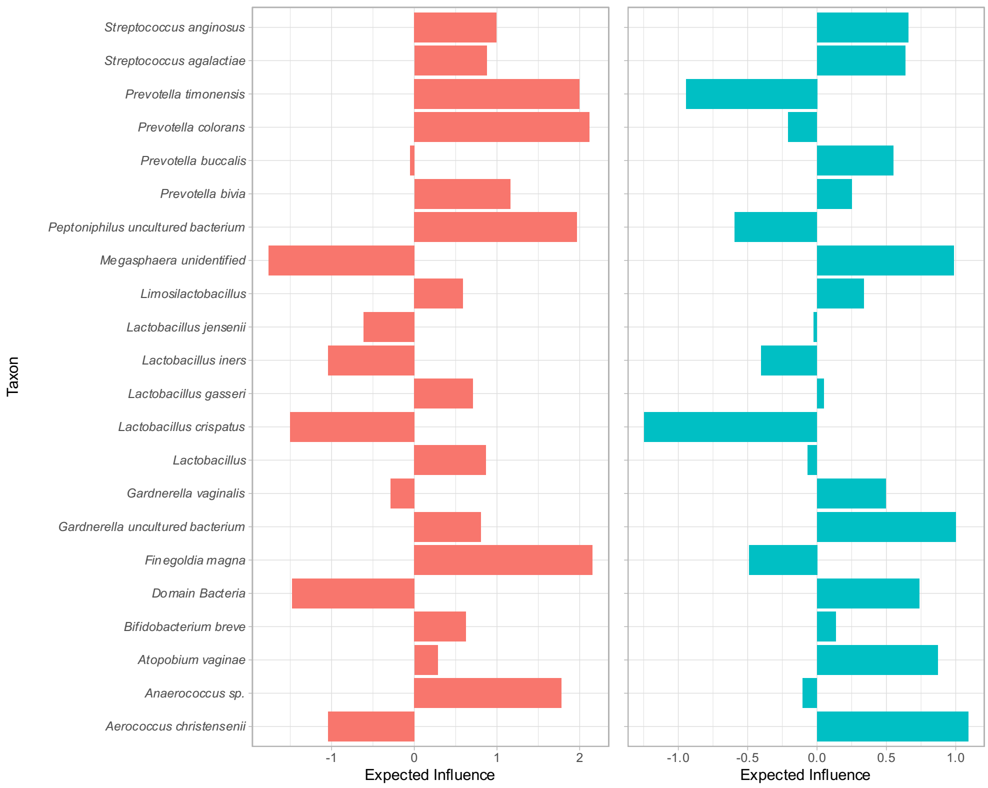

Figure S2. Distribution of nodes with the most positive and negative Expected Influence values (scaled) in non-secretors (red) and secretors (blue) calculated for vaginal microbiome samples taken in early pregnancy.

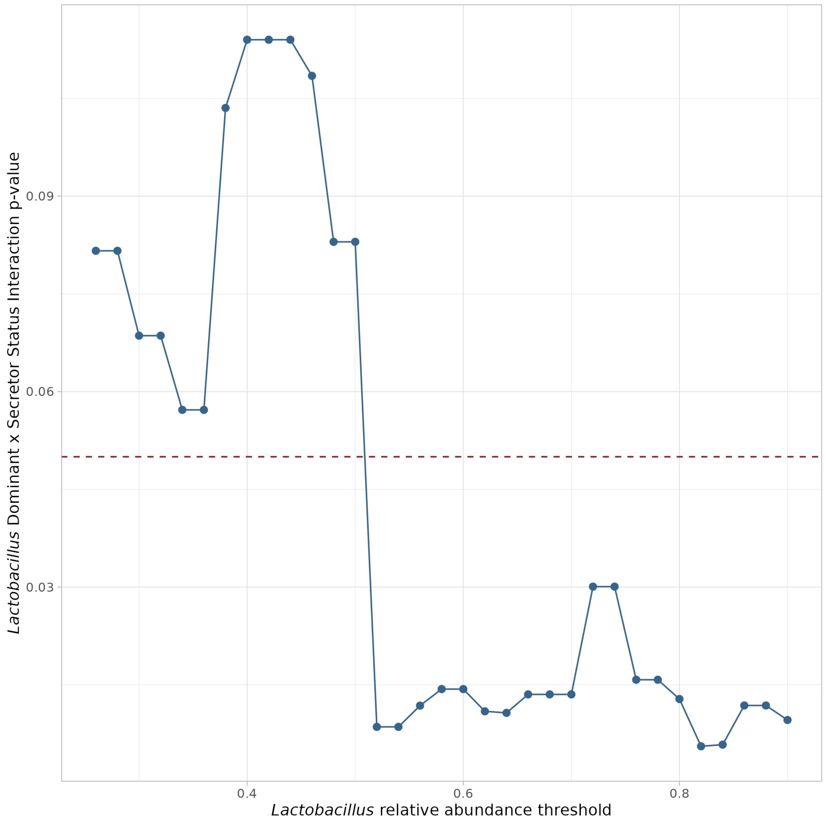

Figure S3. Sensitivity analysis of the impact of *Lactobacillus* relative abundance threshold to separate *L.* Dominated from *L.* Depleted cases on the *p-value* for the Secretor Status x *Lactobacillus* interaction in the early gestational period.

Supplementary Tables

Table S1. Clinical characteristics and inferred secretor status of the study cohort. Sample sizes (number of pregnancies) and percentages (in brackets) are shown. Women (n=302) were sampled up to three times throughout pregnancy (Early: 63 – 131 days [n=263, median of 106], Mid:133 – 178 days [n=264, median of 150] and Late: 183 – 251 [n=238, median of 214]). Secretor status was inferred from the presence of previously documented nonsense and missense mutations (heterozygotes were assumed to be secretors).*1 Preterm birth. *2 Mid trimester loss.

Variable		Secretor (n=219)	Non-secretor (n=83)
Mean±SD gestational length in days		262 ± 27	262 ± 29
Pregnancy outcome	Term (> 37 weeks)	157 (72%)	63 (76%)
	Moderate to late preterm (32-37 weeks)	42 (19%)	11 (13%)
	Very preterm (28-32 weeks)	10 (4.6%)	5 (6.0%)
	Extremely preterm (< 28 weeks)	10 (4.6%)	4 (4.8%)
Mean±SD maternal age in years		32.8 ± 4.7	33.9 ± 5.1
Total number of swabs collected	1	19	9
	2	57	28
	3	143	46
Previous PTB[*1]/MTL[*2]	Yes	95 (43%)	32 (39%)
	No	122 (56%)	46 (55%)
	Unknown	2 (0.9%)	5 (6%)
Previous cervical excisional treatment	Yes	76 (35%)	28 (34%)
	No	143 (65%)	55 (72%)
Cervical stitch	Yes	76 (35%)	24 (29%)
	No	146 (67%)	59 (71%)
BMI	<18.5	4 (1.8%)	0 (0%)
	18.5-24.99	115 (53%)	49 (59%)
	25.0-29.99	63 (29%)	21 (25%)
	>30.0	37 (17%)	13 (16%)
Ethnicity	White	132 (60%)	55 (66%)
	Asian	30 (14%)	11 (13%)
	Black	46 (21%)	16 (19%)
	Arab	5 (2.3%)	1 (1.2%)
	Mixed/Other	6 (2.7%)	0 (0%)

Table S2. Number of samples (swabs) available and outcome distribution per timepoint.

Outcome	Non-secretor			Secretor		
	Early (70)	Mid (72)	Late (61)	Early (192)	Mid (192)	Late (177)
Term	53 (76%)	57 (79%)	50 (82%)	141 (73%)	144 (75%)	133 (75%)
Moderate to late preterm (32-37 weeks)	8 (11%)	8 (11%)	9 (15%)	36 (19%)	34 (18%)	36 (20%)
Very preterm (28-32 weeks)	5 (7.1%)	4 (5.6%)	2 (3.3%)	7 (3.6%)	8 (4.2%)	5 (2.8%)
Extremely preterm (< 28 weeks)	4 (5.7%)	3 (4.2%)	0 (0%)	8 (4.2%)	6 (3.1%)	3 (1.7%)

Table S3. Mean gestational length for secretors and non-secretors and their vaginal microbiomes through pregnancy. Sample sizes are included in brackets.

Microbiome	Early (262)		Mid (264)		Late (238)	
	Secretor (192)	Non-secretor (70)	Secretor (192)	Non-secretor (72)	Secretor (177)	Non-secretor (61)
Lactobacillus dominated	262.52 (151)	266.21 (57)	263.65 (153)	264.27 (60)	266.14 (133)	267.67 (48)
Lactobacillus depleted	266.05 (41)	241.54 (13)	265.41 (39)	258.50 (12)	265.25 (44)	267.62 (13)
CST 1	265.81 (90)	270.46 (30)	266.18 (93)	265.35 (37)	267.76 (83)	266.27 (26)
CST 2	254.70 (10)	0.00 (0)	260.17 (12)	266.00 (1)	249.57 (7)	266.00 (1)
CST 3	258.12 (49)	259.96 (27)	258.35 (48)	261.77 (17)	264.80 (45)	270.11 (19)
CST 4	266.25 (28)	242.11 (9)	268.96 (25)	255.00 (9)	268.59 (27)	268.50 (6)
CST 5	265.07 (15)	277.50 (4)	263.43 (14)	266.13 (8)	261.93 (15)	266.11 (9)

Table S4. Analysis of deviance results from generalised linear mixed effects modelling (GLMM) of gestational length (days) using *Lactobacillus* status (at three timepoints in pregnancy) and ethnicity as a random effect. [†]Significant in the model at a=0.05. *1 Preterm birth. *2 Mid trimester loss.

Model	Variable	Chisq	DF	P-value
Early (n=256)	Maternal age	0.145	1	0.704
	BMI	1.795	1	0.180
	Previous PTB[*1]/MTL[*2]	31.886	1	1.652×10^{-8}[†]
	Cervical stitch	13.879	1	1.950×10^{-4}[†]
	Previous cervical excisional treatment	1.986	1	0.159
	Secretor	0.026	1	0.873
	Lactobacillus	0.205	1	0.650
	Secretor × *Lactobacillus*	6.498	1	0.0108[†]
Mid (n=257)	Maternal age	1.618	1	0.203
	BMI	0.668	1	0.414
	Previous PTB[*1]/MTL[*2]	12.871	1	3.338×10^{-4}[†]
	Cervical stitch	24.003	1	9.620×10^{-7}[†]
	Previous cervical excisional treatment	4.291	1	0.038[†]
	Secretor	0.002	1	0.963
	Lactobacillus	2.005	1	0.157
	Secretor × *Lactobacillus*	1.251	1	0.263
Late (n=232)	Maternal age	2.541	1	0.111
	BMI	0.380	1	0.537
	Previous PTB[*1]/MTL[*2]	12.058	1	5.157×10^{-4}[†]
	Cervical stitch	6.857	1	8.828×10^{-3}[†]
	Previous cervical excisional treatment	1.818	1	0.178
	Secretor	0.144	1	0.704
	Lactobacillus	0.716	1	0.394
	Secretor × *Lactobacillus*	0.113	1	0.737

Table S5. Analysis of deviance results from GLMMs of gestational length in days using two different definitions of *L.* Dominated vs *L.* Depleted status based on VALENCIA CSTs, or relative abundance of Genus *Lactobacillus* counts as a continuous covariate. All estimates reported are from models fitted on the early gestation timepoint. †Significant in the model at a=0.05. *1 Preterm birth. *2 Mid trimester loss.

L. Dominated definition	Variable	Chisq	DF	P-value
**VALENCIA CST**	Maternal age	0.252	1	0.615
	BMI	1.291	1	0.256
L. Dominated:	Previous PTB^{*1}/MTL^{*2}	29.727	1	$4.974\times10^{-8\dagger}$
CST I, II, III, V	Cervical stitch	13.076	1	$2.991\times10^{-4\dagger}$
L. Depleted: All CST IV	Previous cervical excisional treatment	2.225	1	0.136
	Secretor	0.067	1	0.797
	Lactobacillus	0.272	1	0.602
	Secretor × *Lactobacillus*	1.797	1	0.180
**Modified VALENCIA CST**	Maternal age	0.248	1	0.619
	BMI	1.150	1	0.284
Same as above, but	Previous PTB^{*1}/MTL^{*2}	28.523	1	$9.260\times10^{-8\dagger}$
IV-C3 (*B. breve)* and	Cervical stitch	12.084	1	$5.086\times10^{-4\dagger}$
other IV cases where a *Lactobacillus sp*. is	Previous cervical excisional treatment	2.293	1	0.130
the majority taxon are	Secretor	0.052	1	0.819
classified as *L.*	*Lactobacillus*	0.032	1	0.858
Dominated	Secretor × *Lactobacillus*	1.820	1	0.177
***Lactobacillus* %**	Maternal age	0.177	1	0.674
Relative abundance of	BMI	1.520	1	0.218
Lactobacillus Genus	Previous PTB^{*1}/MTL^{*2}	30.207	1	$3.884\times10^{-8\dagger}$
counts as a	Cervical stitch	12.667	1	$3.721\times10^{-4\dagger}$
continuous covariate	Previous cervical excisional treatment	2.261	1	0.133
	Secretor	0.022	1	0.881
	Lactobacillus %	0.126	1	0.723
	Secretor × *Lactobacillus %*	5.098	1	$0.024^{\dagger}$

Table S6. Unstandardised coefficients (*b*) and 95% confidence intervals (CI) from gamma generalised linear mixed effects modelling (GLMM) of gestational length (in days) using two different definitions of *L.* Dominated vs *L.* Depleted status based on VALENCIA CSTs, or relative abundance of Genus *Lactobacillus* counts as a continuous covariate. All estimates reported are from models fitted on the early gestation timepoint. *[1] Preterm birth. *[2] Mid-trimester loss. * p<0.05. ** p<0.01. *** p<0.001.

	VALENCIA<br>β (95% CI)	Modified VALENCIA CST<br>β (95% CI)	*Lactobacillus* %<br>β (95% CI)
Intercept	3.285 (2.512, 4.058) ***	3.264 (2.498, 4.031) ***	3.146 (2.365, 3.927) ***
Maternal age	0.004 (-0.012, 0.021)	0.004 (-0.012, 0.021)	0.003 (-0.013, 0.020)
BMI	-0.011 (-0.030, 0.008)	-0.010 (-0.029, 0.008)	-0.012 (-0.030, 0.007)
Previous PTB*[1]/MTL*[2]	0.533 (0.342, 0.725) ***	0.525 (0.332, 0.718) ***	0.530 (0.341, 0.719) ***
Cervical stitch	0.353 (0.162, 0.545) ***	0.340 (0.148, 0.531) ***	0.347 (0.156, 0.537) ***
Previous cervical excisional treatment	-0.149 (-0.345, 0.047)	-0.151 (-0.347, -0.045)	-0.148 (-0.342, 0.045)
Secretor × *Lactobacillus*	0.386 (-0.178, 0.949)	0.486 (-0.220, 1.192)	-0.808 (-1.509, -0.107) *

Table S7. Analysis of deviance results from GLMMs of gestational length in days using Community State Type (CST) (with ethnicity as a random effect) highlighting covariates that are significant explanatory variables in the models. †Significant in the model at α=0.05. *1 Preterm birth. *2 Mid trimester loss.

Model	Variable	Chisq	DF	P-value
Early (n=256)	Maternal age	0.432	1	0.511
	BMI	1.425	1	0.233
	Previous PTB*1/MTL*2	27.490	1	$1.579 \times 10^{-7\dagger}$
	Cervical stitch	14.141	1	$1.696 \times 10^{-4\dagger}$
	Previous cervical excisional treatment	2.314	1	0.128
	Secretor	0.065	1	0.798
	CST	1.941	4	0.747
	Secretor × CST	4.602	3	0.203
Mid (n=257)	Maternal age	2.118	1	0.146
	BMI	1.135	1	0.287
	Previous PTB*1/MTL*2	12.658	1	$3.739 \times 10^{-4\dagger}$
	Cervical stitch	23.282	1	$1.399 \times 10^{-6\dagger}$
	Previous cervical excisional treatment	4.595	1	0.0321†
	Secretor	0.0001	1	0.992
	CST	4.520	4	0.340
	Secretor × CST	3.766	4	0.439
Late (n=232)	Maternal age	2.041	1	0.153
	BMI	0.495	1	0.482
	Previous PTB*1/MTL*2	10.793	1	$1.019 \times 10^{-3\dagger}$
	Cervical stitch	7.559	1	$5.972 \times 10^{-3\dagger}$
	Previous cervical excisional treatment	2.185	1	0.139†
	Secretor	0.164	1	0.686
	CST	4.761	4	0.313
	Secretor × CST	0.706	4	0.951

Table S8. Unstandardised coefficients (*b*), and 95% confidence intervals (CI) from GLMMs of gestational length and incorporating ABO status (as an additional independent variable). Blood group A, Secretors with *Lactobacillus* dominated microbiota are baseline in the model. *[1] Preterm birth. *[2] Mid-trimester loss. * p<0.05. ** p<0.01. *** p<0.001.

	Early (n=197)	Mid (n=195)	Late (n=175)
	β (95% CI)	β (95% CI)	β (95% CI)
Intercept	3.567 (2.635, 4.499) ***	3.320 (2.385, 4.256) ***	2.929 (1.967, 3.892) ***
Maternal age	0.002 (-0.018, 0.022)	0.005 (-0.015, 0.024)	0.011 (-0.010, 0.031)
BMI	-0.021 (-0.043, 0.001)	-0.018 (-0.038, 0.002)	-0.010 (-0.030, 0.010)
Previous PTB*[1]/MTL*[2]	0.578 (0.350, 0.805) ***	0.398 (0.172, 0.624) ***	0.300 (0.071, 0.530) *
Cervical stitch	0.363 (0.130, 0.597) **	0.454 (0.255, 0.653) ***	0.283 (0.079, 0.487) **
Previous cervical excisional treatment	-0.100 (-0.325, 0.126)	-0.075 (-0.306, 0.155)	-0.062 (-0.288, 0.164)
ABO (AB)	-0.179 (-0.672, 0.314)	0.041 (-0.438, 0.520)	0.072 (-0.379, 0.524)
ABO (B)	0.188 (-0.095, 0.470)	0.397 (0.126, 0.669) **	0.309 (0.031, 0.588) *
ABO (O)	-0.057 (-0.277, 0.163)	-0.021 (-0.230, 0.188)	-0.025 (-0.242, 0.191)
Secretor × *Lactobacillus*	0.690 (0.161, 1.220) *	0.103 (-0.439, 0.645)	-0.051 (-0.591, 0.489)
